# Evaluation of infectious titer in a candidate HSV type 2 vaccine by a quantitative molecular approach

**DOI:** 10.1186/1471-2180-13-284

**Published:** 2013-12-06

**Authors:** Ali Azizi, Mei Tang, Lucy Gisonni-Lex, Laurent Mallet

**Affiliations:** 1 Microbiology & Virology Platform, Department of Analytical Research & Development North America, Sanofi Pasteur, 1755 Steeles Avenue West, Toronto, Ontario M2R 3 T4, Canada

**Keywords:** Infectious titre, HSV type 2, RT-qPCR, Vaccine, Potency

## Abstract

**Background:**

One of the critical tasks in analytical testing is to monitor and assign the infectivity or potency of viral based vaccines from process development to production of final clinical lots. In this study, a high throughput RT-qPCR based approach was developed to evaluate the infectious titre in a replication-defective HSV-2 candidate vaccine, called HSV529. This assay is a combination of viral propagation and quantitative RT-PCR which measures the amount of RNA in infected cells after incubation with test samples.

**Results:**

The relative infectious titre of HSV529 candidate vaccine was determined by a RT-qPCR method targeting HSV-2 gD2 gene. The data were analyzed using the parallel-line analysis as described in the European Pharmacopoeia 8^th^ edition. The stability of HSV529 test samples were also investigated in a concordance study between RT-qPCR infectivity assay and a classical plaque assays. A suitable correlation was determined between both assays using an identical sample set in both assays. The RT-qPCR infectivity assay was further characterized by evaluating the intermediate precision and accuracy. The coefficient of variation from the six independent assays was less than 10%. The accuracy of each of the assay was also evaluated in the range of 92.91% to 120.57%.

**Conclusions:**

Our data demonstrate that the developed RT-qPCR infectivity assay is a rapid high throughput approach to quantify the infectious titer or potency of live attenuated or defective viral-based vaccines, an attribute which is associated with product quality.

## Background

The conventional *in-vitro* assays to measure the titer or potency of live viral-based vaccines are usually based on the infectivity of the vaccine virus in cell cultures (plaque assay or CCID50) [1-5]. In both methods, the experiment duration is long due to the time needed for virus replication producing the biological effect. In addition, there is a cell substrate limitation with the traditional methods, and only viruses that cause a detectable biological effect on infected cells can be evaluated.

The introduction of real time PCR technology for the quantitation of viral infectivity has significantly improved viral infectivity assays. This method is a combination of virus propagation and quantitative PCR (qPCR) or RT-qPCR. In a study by Ranheim et al., [6] a RT-qPCR assay was developed to detect rotavirus vaccine (Rota Teq) infectivity within two days. In this assay, the confluent Vero cells in 96-well plates were inoculated with serial dilutions of test samples, a pentavalent reassortant rotavirus reference standard, and assay controls. After 24 hours, Vero cells were lysed and the lysates were measured by RT-qPCR to quantify viral replication. In another study, Schalk et al., [4] developed a rapid assay for the measurement of infectivity-potency in MMR trivalent vaccines based on a qPCR infectivity assay. The assay was able to demonstrate the potency of mumps and measles viruses within a period of 2 days. Since rubella virus replicates slower than measles and mumps, the potency estimation for rubella virus was PCR-based assays as end-points since a plaque assay for measles and rubella virus usually takes 9 days [4]. This period of time for detection of mumps virus in cell line is 6 days. A one week time reduction in the qPCR infectivity assay without loss of precision compared to a plaque assay and TCID50 was a major advantage of the assay.

Dr. Knipe’s group at Harvard Medical School constructed a candidate Herpes Virus vaccine through deletion of the UL5 and UL29 coding regions of HSV-2 virus [7]. The resultant vaccine, HSV529, is being developed by Sanofi Pasteur and is currently under a human phase I clinical trial [8,9]. The AV529-19 cell line is used for the propagation of HSV529. This cell line is a Vero-based cell line specifically engineered to express the HSV-1 UL5 and UL29 transgenes. With expression of the HSV-1 UL5 and UL29 genes, AV529-19 is able to support replication of HSV529 [8,9]. Herein, we have developed a high throughput RT-qPCR-based approach for evaluation of the infectious titer of HSV529 candidate vaccine. The developed infectivity RT-qPCR based approach determines relative quantification to an appropriately constructed in-house reference control. The assay’s accuracy and intermediate precision was also investigated to ensure suitable performance of this analytical method. Furthermore, a concordance stability study between the developed method and a classical plaque assay was performed to investigate the correlation between both assays. The results obtained from both assays using the same identical sample set demonstrated a suitable linear correlation between both approaches. In summary, the developed RT-qPCR infectivity assay is a rapid method with high-throughput capacity that can be applied to quantify the infectious titer of HSV529 candidate vaccine. This approach could also be applied to other live or attenuated viral vaccines to quantify the infectious titer of product.

## Results

### Specificity of HSV-2 various target genes and optimization of harvest time

**Figure 1:**
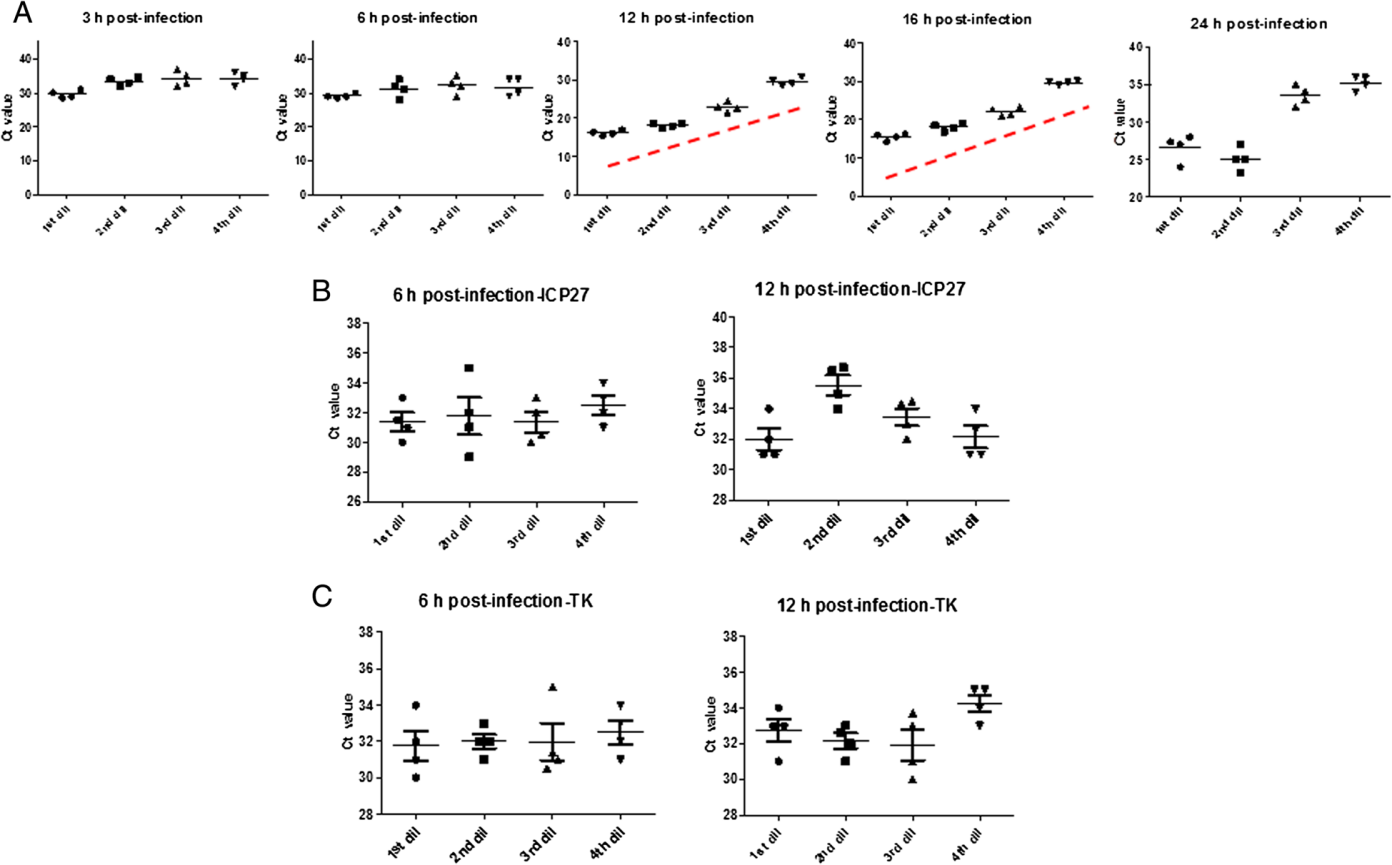
**The accumulation of HSV529 RNA after post-infection.** The infected cells were lysed after each time point (3, 6, 12, 16, and 24 hours post-infection), RNA was purified followed by DNase treatment, and RT-qPCR performed using specific primers for HSV-2. **A**. The accumulation of HSV529 targeting gD2 gene is shown. A linear relationship between the logarithm of the HSV529 concentration and the *C*_*T*_ values were observed at 12 to 16 h post-infection. **B**. The accumulation of HSV529 targeting ICP-27 gene is shown No linear relationship between the logarithm of the HSV529 concentration and the *C*_*T*_ values were observed at any time points (3, 6, 12, 16, and 24 h). As a representative, the accumulation of HSV529 RNA targeting ICP-27 after 6 h and 12 h post-infection is shown. **C**. The accumulation of HSV529 RNA targeting TK gene after 3 h and 6 h post-infection is shown. No linear relationship between the logarithm of the HSV529 concentration and the *C*_*T*_ values were observed at any time points (3, 6, 12, 16, and 24 h). dil:dilution.

The accumulation of HSV529 RNA during infection was measured by one step RT-qPCR at 3, 6, 12, 16, and 24 hours post-infection using specific primers for ICP27, TK, and gD2. A sufficient quantity of RNA from cells infected with HSV529 was extracted by adding 50 μl of each HSV529 dilution to each well in a 96-well plate format. The cells were lysed, RNA was purified, DNase treated, and one-step RT-qPCR was performed. After RT-qPCR, *C*_*T*_ values of each targeted gene were plotted versus time post infection. No trends were observed for plots of *C*_*T*_ versus HSV529 concentration for studies targeting ICP27 or TK genes 3–24 hours post-infection (Figure 1B and 1C). However, one-step RT-qPCR using gD2 primers showed a linear relationship between the logarithm of the viral concentration and the *C*_*T*_ values 12–16 hr post-infection. The slope of the graph flattens, and no trends were observed 24 hours post-infection as replication of HSV529 virus, causes death of AV529-19 cells over time. The accumulation of HSV529 viral concentration during infection at 3, 6, 12, 16, and 24 hours post-infection using specific gD2 primers is shown in Figure 1A. The overall results show that HSV-2 gD2 is a suitable targeted gene for evaluation of HSV529 infectious titre 12–16 hour post-infection.

### Evaluate the infectivity of HSV529 test samples by targeting HSV-2 gD2 gene

**Figure 2:**
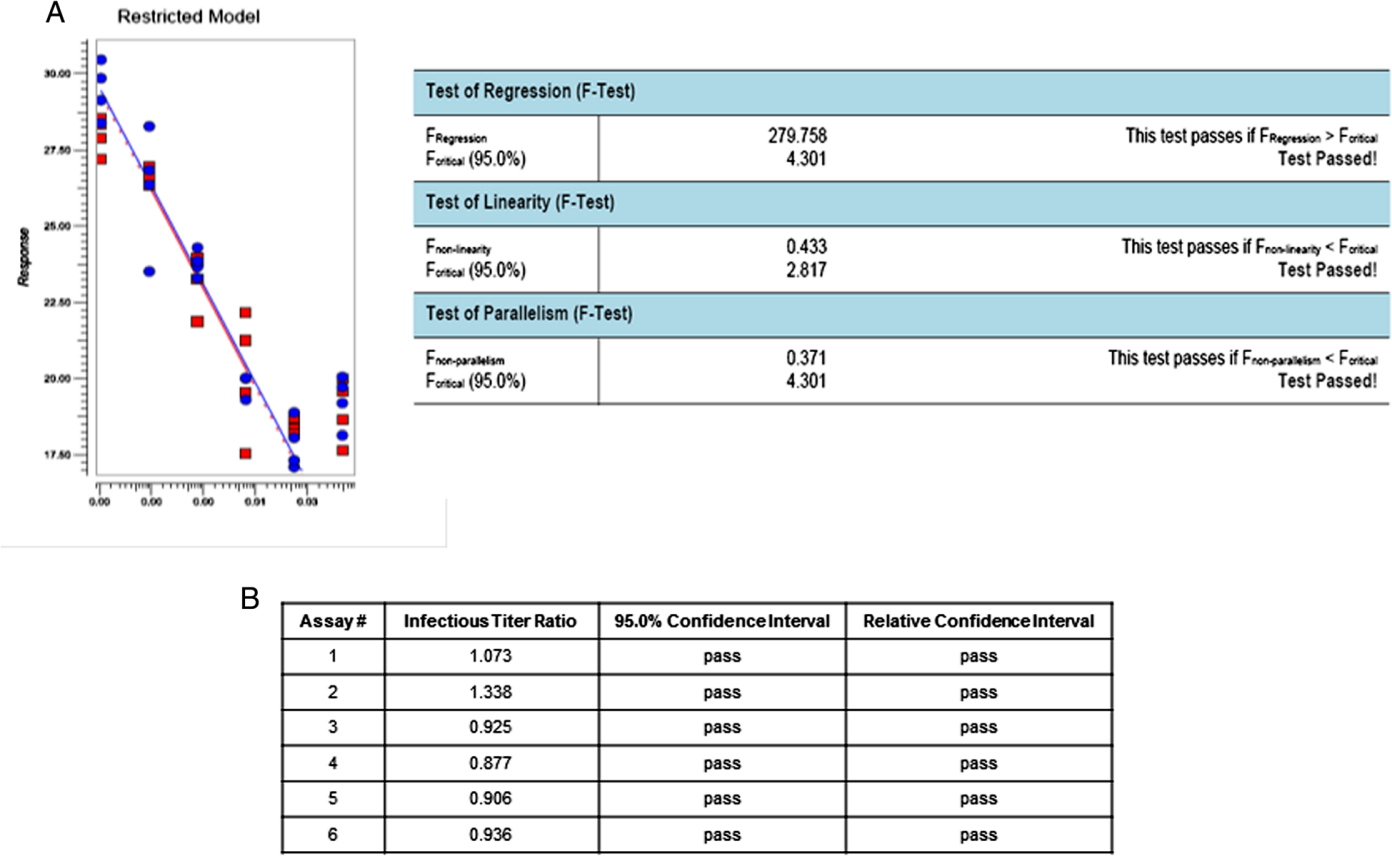
**The infectious titer ratios from six independent assays using the same lot of HSV529 as the in-house reference control and the test sample. A**. PLA analysis and acceptance criteria from one representative assay. **B**. The infectious titer ratio, 95% confidence interval, and relative confidence interval observed for the six independent assays are shown.

**Figure 3:**
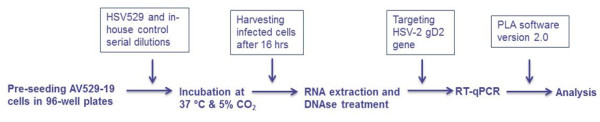
**Overview of the developed RT-qPCR infectivity assay.** Ninety six well plates were pre-seeded with AV529-19. Next day, cells were infected with the serial dilutions of HSV529 or in-house reference control. RNA was extracted 16 hours post infection, treated with DNase, RT-qPCR performed targeting HSV-2 gD2, and infectious titer assigned by PLA analysis.

The assay targeting gD2 was performed six times in a 96-well plate format and the results were analyzed through extrapolation or PLA software 2.0. Briefly, 96-well plates were seeded with AV529-19 a day before infection. Next day, cells were infected with the serial dilutions of HSV529 (5 dilutions with 4 replicates for each dilution). The same lot of HSV529 was used as both test sample and in-house reference control in all six independent assays. RNA was extracted 16 hours post infection, treated with DNase, RT-qPCR performed targeting HSV-2 gD2, and infectious titer assigned by PLA analysis. Since the same HSV529 lot was used as the test sample and the in-house reference control, it was expected to observe two close parallel lines (infectious titer ratio of ~1.0) after PLA analysis. The infectious titer ratio, 95% confidence interval, and relative confidence interval observed for the six independent assays are shown in Figure 2. A simplified diagram from the developed RT-qPCR infectivity assay targeting HSV-2 gD2 gene is shown in Figure 3.

### A comparative stability study between RT-qPCR infectivity assay and a classical plaque assay

**Figure 4:**
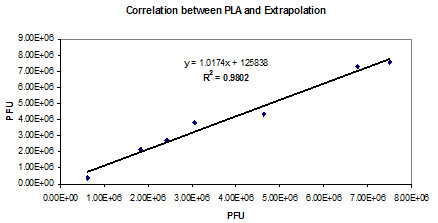
**Correlation between the results analyzed by extrapolation or PLA.** The infectious titer was evaluated by RT-qPCR and the results were analyzed by both extrapolation and PLA.

To determine if RT-qPCR infectivity assay is a suitable approach to evaluate the stability of HSV529 test samples, a concordance study between the RT-qPCR infectivity assay and a plaque assay was conducted using identical test samples set in both assays. HSV529 test samples were incubated at 4–8°C or 22–25°C in various time points and the infectious titre was measured by a classical plaque assay. These samples set were also tested by the RT-qPCR infectivity assay and the results were analyzed by extrapolation or PLA. A suitable correlation was observed between PLA or extrapolation analysis (Figure 4). A suitable correlation was also determined between the infectious titer as measured by RT-qPCR infectivity assay or plaque assay (Table 1).

**Table 1:** Infectious titre results obtained by RT-qPCR infectivity assay or plaque assay

**A.**					
	**0-1 hr**	**1 day**	**3 days**	**6 days**	**7 days**
**RT-qPCR infectivity**	7.50E + 06	7.28E + 06	4.35E + 06	3.35E + 06	2.43E + 06
**Plaque assay**	7.36E + 06	5.55E + 06	4.52E + 06	4.43E + 06	2.70E + 06
**B.**					
**RT-qPCR infectivity**	3.06E + 06	1.14E + 06	2.14E + 06	1.30E + 06	3.78E + 05
**Plaque assay**	3.23E + 06	3.40E + 06	2.80E + 06	1.55E + 06	N/A

HSV529 test samples were incubated at A. 4–8°C or B. 22-25°C at various time points and the infectious titre was measured by RT-qPCR infectivity assay or plaque assay.

### Evaluation of intermediate precision and accuracy in the developed RT-qPCR infectivity assay

To evaluate the intra-laboratory variation and closeness of data, the intermediate precision and accuracy of the developed RT-qPCR infectivity assay was assessed. For this purpose, the HSV529 in-house reference control was used as both test sample and in-house reference control. As described, AV529-19 cells were infected and the total RNA was extracted and processed 16 hours post-infection. RT-qPCR was performed targeting gD2 gene, and the results were analyzed through PLA software version 2.0. The assay was performed six times by two analysts on different days over a period of two months. The coefficient of variation (%CV) from the six independent assays was 9.19%. The accuracy of the assay was calculated by evaluating the percentages of values obtained by RT-qPCR infectivity assay versus the expected infectious titre values by plaque assay (1.41 × 10^7^ pfu/ml). The accuracy of assay was evaluated in the range of 92.91% to 120.57% (Table 2).

**Table 2:** The intermediate precision and accuracy of the developed RT-qPCR infectivity assay is determined

**Assay #**	**RT-qPCR (pfu)**	**RT-qPCR (log_pfu)**	**Plaque assay**	**Accuracy%**	**CV%**
			**(Mean from 30 assays)**		
1	1.50E + 07	16.52	1.41E + 07	106.38	
2	1.63E + 07	16.60		115.60	
3	1.45E + 07	16.48		102.34	
4	1.70E + 07	16.64		120.57	
5	1.54E + 07	16.54		109.22	
6	1.31E + 07	16.38		92.91	9.19

RT-qPCR infectivity assay was performed six times by two analysts on different days. The accuracy of the assay was calculated by evaluating the percentages of values obtained by RT-qPCR infectivity assay versus the expected infectious titre values by plaque assay. The CV% from the six independent assays is also determined.

## Discussion

There are several challenges with conventional *in-vitro* assays (plaque or CPE) to measure the titer of live attenuated or defective viral-based vaccines [4,6]. The traditional assays are usually laborious and sometimes hard to interpret. The time due to-assay-completion and cell substrate limitations are also challenges with the conventional *in-vitro* assays. For instance, it takes nine days to measure the infectious titre in measles or rubella vaccines [4]. Furthermore, traditional methods require virus neutralization for characterization of infectivity or potentially potency in multivalent viral vaccines. However, a PCR-infectivity based approach does not require virus neutralization, making it a more attractive alternative for multivalent viral vaccines. Although HSV529 candidate vaccine has not been faced with some of these challenges (the HSV-2 virus is able to form plaques in AV529-19 cells over 3 days and is not a multivalent vaccine), a RT-qPCR infectivity based-approach was developed to enhance the assay’s throughput (testing more samples in a shorter time).

During HSV-2 replication, the five viral genes expressed in the immediate-early (phase α), encode regulatory proteins [10,11]. After the immediate-early step, early genes are activated (phase β), and these encode proteins required for replication of the viral genome. After genome replication in the early phase, the late step (phase γ) occurs, where HSV-2 structural proteins are expressed and the virus is formed [10,11]. One of the critical features of the RT-qPCR infectivity assay was to determine the specificity of the assay targeting appropriate HSV-2 gene. Therefore, one gene (ICP27, TK, and gD2) from each of the replication phases was targeted. We were able to observe a linear relationship between the logarithm of the HSV529 concentration and the *C*_*T*_ values by targeting the gD2 gene and not the ICP27 or TK genes. It has to be noted that during the late gD2 expression, the immediate-early and early proteins are also generated and the full form of the virus is completed. HSV-2 gD2 RNA accumulation starts to level off approximately 12 hours post-infection and remains relatively steady for up to 16 hour post-infection.

The developed assay is a combination of *in-vitro* HSV529 propagation in the suitable cell line for a short HSV-2 replication cycle followed by a RT-qPCR. The infectious titers of the test samples are estimated relative to an in-house reference control. This in-house reference control was titrated in the lab using conventional plaque assay and validated based on 30 independent assays accordance to the International Conference on Harmonisation (ICH) guideline [12]. Therefore, the assay measures the relative infectious unit based on the in-house reference control unitage. Briefly, confluent AV529 cells in 96-well plates were inoculated with serial dilutions of HSV529 test samples and an HSV529 in-house control, to produce a standard curve followed by incubation for 16 hours. Thereafter, HSV529-19 cells were lysed, total RNA purified, DNAse treated, and RT-qPCR targeting HSV-2 gD2 gene was performed to quantitate HSV529 nucleic acid produced during replication. The relative infectious titre for each sample was determined using the parallel-line analysis as described in the European Pharmacopoeia 8.0 [13]. The analysis by extrapolation is not an appropriate approach as several parameters including the similar conditions between the in-house reference control and test samples are not considered during analysis. In this study, the correlation between test samples and the in-house reference control was assessed using PLA software version 2.0. Before PLA analysis, all *C*_*T*_ values for the in-house reference control and test samples were subjected to standard outlier analysis, with the limit that no more than one data point (one replicate out of the four replicates) per HSV529 dilution could be removed. Afterwards, each assay was analyzed by PLA software. The assay was considered valid if the regression, linearity, and parallelism were significant.

To investigate if RT-qPCR infectivity assay is a suitable method to evaluate the stability of HSV529 test samples, a concordance study was conducted between the RT-qPCR infectivity assay and a conventional infectivity plaque assay using identical test samples. While the results illustrated a suitable correlation (R2 ~0.91) between the qRT-PCR infectivity assay and the plaque assay, higher cost and complexity of RT-qPCR infectivity assay were two drawbacks of this method compare to a traditional method.

To evaluate the closeness of the analytically determined HSV529 infectious titre values, the accuracy of the method was evaluated in six independent assays by two analysts on different days. The accuracy was determined as the percentage of the infectious titre values obtained by RT-qPCR versus infectious titre values by a plaque assay. The accuracy was evaluated in the range of 92.91% to 120.57%, indicating a suitable accuracy for the assay. The intermediate precision of the assay was also evaluated to measure the variation of the obtained data. To evaluate this parameter, the assay was performed six times by two different operators over a time period of 2 months. The mean value of this run control was 16.53 log pfu/ml with a standard deviation of 0.091, resulting in a coefficient of variation of 9.19.

## Conclusions

In this study, a RT-qPCR based approach was utilized to specifically detect and quantitate the HSV529 RNA after productive infection in AV529-19 cells. The results show that the developed RT-qPCR infectivity assay is a reproducible approach that can quantitate the HSV529 infectious titre before the plaque assay formation is visible on day 3. The described RT-qPCR infectivity approach might also be a suitable approach for determination of potency of test samples, however; further evaluation of sub-potent lots and/or assessing clinical data is required.

## Methods

### Plaque assay

The infectious titre of an HSV529 (lot#10954) was determined through a plaque assay on AV529 cells by performing 30 independent plaque assays. This lot was determined as the HSV529 in-house reference control. Briefly, serial dilutions of the viral material was allowed to adsorb on the AV529 cell monolayers at 36°C ± 1°C, 5% ± 2% after which the volume of infection media was adjusted to a suitable volume to allow for incubation at 36°C ± 1°C, 5% ± 2% for 48 hours. After the 48 hour incubation step, the cell monolayers were fixed and stained with a crystal violet (Sigma) and methanol stain and the visible plaques were enumerated by eye and used to assign a titre in log_10_ pfu/ml. The assigned mean infectious titre from 30 independent assays was 1.41 × 10^7^ pfu/ml.

### Cell culture and infection

AV529-19 cells were cultured in DMEM/F12 (Sigma) supplemented with 1% (v/v) Penicillin**/**Streptomycin (Sigma), 1% heat inactivated ultra-low IgG-FBS (Invitrogen), 1% L-glutamine (Sigma), and maintained in a 37°C incubator in 5% CO_2._ Prior to each assay, cells were plated one day in advance in 96-well tissue culture plates (Becton Dickinson) at a density of 4×10^4^ cells per well in a volume of 200 μl. Next day, plates were visually inspected under a microscope to confirm the cell monolayer was 80-100% confluent. Serial dilutions of the HSV529 test samples as well as the HSV529 in-house reference control were prepared in culture media. The media from each well was removed, and 50 μl of each viral dilution was added to each well (four replicates were used for each dilution). Afterwards, 50 μl media was dispensed into each infected well for a total volume of 100 μl. Afterwards, 100 μl media was added to the uninfected and negative control wells. The plates were placed at 36 ± 1°C, 5% CO_2_ incubator for 16 hours.

### RNA isolation

Total RNA was isolated using total RNA purification 96-well kit (Norgen Biotek). The purified RNA was treated with TURBO DNA-free kit (Applied Biosystems) according to manufacture’s instruction.

### Quantitative real-time RT-PCR (RT-qPCR)

The RT-qPCR was performed by targeting the HSV-2 immediate early (ICP27), early (TK) and late (gD2) genes. For ICP27, the forward and reverse primers were 5′- GCC ACT CTC TTC CGA CAC -3′ and 5′- CAA GAA CAT CAC ACG GAA C-3′, respectively. For TK, the forward and reverse primers were 5′-TGG ATT ACG ATC AGT CGC C -3′ and 5′-ACA CCA CAC GAC AAC AAT GC-3′, respectively. For gD2, the forward and reverse primers were 5′-TCA GCG AGG ATA ACC TGG GA-3 and 5′-GGG AGA GCG TAC TTG CAG GA-3, respectively. The ICP27, TK, and gD2 primers have been previously described and tested in other studies. [14-16]. All the primers were purchased from Life Biotechnologies. One step RT-qPCR was performed using SYBR Green PCR master mix (Applied Biosystems), MultiScribe Reverse Transcriptase (50 U/μl, Applied Biosystems), RNase Inhibitor (20 U/μl, Applied Biosystems), 1 pmol of each forward and reverse primer, and 2 μl isolated RNA in a total volume of 25 μl. The RT-qPCR program consisted of 30 min reverse transcription reaction at 48°C, 10 min denaturation at 95°C, 40 cycles of 15 seconds denaturation at 95°C, 1 min annealing at 60°C and extension at 95°C for 15 seconds. The assay was performed using the Mastercycler® ep realplex (Eppendorf).

### Data analysis

The data from the qRT-PCR infectivity assay were analyzed by the extrapolation statistical approach using Eppendorf Mastercycler Software (Applied Biosystems) or Parallel-Line Analysis (PLA) using the PLA software version 2.0.

## Competing interests

The authors declare that they have no competing interests.

## Authors’ contributions

AA designed the study, performed the experiments, and drafted the manuscript. MT performed the statistical analysis. LG and LM participated in the design of the study and assisted in revising the manuscript. All authors read and approved the final manuscript.

## References

[B1] Minagawa T Sakuma T Kuwajima S Yamamoto TK Iida H Characterization of measles viruses in establishment of persistent infections in human lymphoid cell line J Gen Virol 1976 13 3 361 379 10.1099/0022-1317-33-3-361 794440

[B2] Wadey CN Faragher JT Australian infectious bronchitis viruses: plaque formation and assay methods Res Vet Sci 1981 13 1 66 69 6264555

[B3] Beales LP Wood DJ Minor PD Saldanha JA A novel cytopathic microtitre plate assay for hepatitis A virus and anti-hepatitis A neutralizing antibodies J Virol Methods 1996 13 1–2 147 154 8793842 10.1016/0166-0934(96)02035-6

[B4] Schalk JA de Vries CG Jongen PM Potency estimation of measles, mumps and rubella trivalent vaccines with quantitative PCR infectivity assay Biologicals 2005 13 2 71 79 10.1016/j.biologicals.2005.01.001 15939284

[B5] Sood DK Aggarwal RK Kumar S Sokhey J A rapid test for measuring the infectivity of Yellow Fever vaccine Vaccine 1995 13 5 427 428 10.1016/0264-410X(94)00035-L 7639009

[B6] Ranheim T Mathis PK Joelsson DB Development and application of a quantitative RT-PCR potency assay for a pentavalent rotavirus vaccine (RotaTeq) J Virol Methods 2006 13 2 193 201 10.1016/j.jviromet.2005.08.013 16214228

[B7] Da CX Kramer MF Zhu J Brockman MA Knipe DM Construction, phenotypic analysis, and immunogenicity of a UL5/UL29 double deletion mutant of herpes simplex virus 2 J Virol 2000 13 17 7963 7971 10.1128/JVI.74.17.7963-7971.2000 10933704 PMC112327

[B8] Delagrave S Hernandez H Zhou C Immunogenicity and efficacy of intramuscular replication-defective and subunit vaccines against herpes simplex virus type 2 in the mouse genital model PLoS One 2012 13 10 e46714 10.1371/journal.pone.0046714 23071620 PMC3469653

[B9] Mundle ST Hernandez H Hamberger J High-purity preparation of HSV-2 vaccine candidate ACAM529 is immunogenic and efficacious in vivo PLoS One 2013 13 2 e57224 10.1371/journal.pone.0057224 23468943 PMC3582571

[B10] Smiley JR Herpes simplex virus virion host shutoff protein: immune evasion mediated by a viral RNase? J Virol 2004 13 3 1063 1068 10.1128/JVI.78.3.1063-1068.2004 14722261 PMC321390

[B11] Manservigi R Argnani R Marconi P HSV recombinant vectors for gene therapy Open Virol J 2010 13 123 156 20835362 10.2174/1874357901004010123 PMC2936037

[B12] Validation of Analytical Procedures, the International Conference on Harmonisation 2005

[B13] Chapter 5.3, Statistical analysis of results of biological assays and tests, European Pharmacopoeia 2013 8

[B14] Da Costa X Jones CA Knipe DM Immunization against genital herpes with a vaccine virus that has defects in productive and latent infection Proc Natl Acad Sci USA 1999 13 12 6994 6998 10.1073/pnas.96.12.6994 10359827 PMC22033

[B15] Haynes JR Arrington J Dong L Braun RP Payne LG Potent protective cellular immune responses generated by a DNA vaccine encoding HSV-2 ICP27 and the E. coli heat labile enterotoxin Vaccine 2006 13 23 5016 5026 10.1016/j.vaccine.2006.03.046 16621198

[B16] Hoshino Y Dalai SK Wang K Comparative efficacy and immunogenicity of replication-defective, recombinant glycoprotein, and DNA vaccines for herpes simplex virus 2 infections in mice and guinea pigs J Virol 2005 13 1 410 418 10.1128/JVI.79.1.410-418.2005 15596834 PMC538700

